# Human Immunodeficiency Virus Type-1 (HIV-1) Continues to Evolve in Presence of Broadly Neutralizing Antibodies More than Ten Years after Infection

**DOI:** 10.1371/journal.pone.0044163

**Published:** 2012-08-30

**Authors:** Antoine Chaillon, Martine Braibant, Stéphane Hué, Samia Bencharif, David Enard, Alain Moreau, Assia Samri, Henri Agut, Francis Barin

**Affiliations:** 1 Université François Rabelais, Inserm UMR 966, Tours, France; 2 CHU Bretonneau, Laboratoire de Virologie, CHU Bretonneau, Tours, France; 3 Centre for Medical Molecular Virology, University College London, London, United Kingdom; 4 Laboratoire Ecologie et Evolution CNRS UMR 7625- Ecole Normale supérieure, Paris, France; 5 Université Pierre et Marie Curie, Inserm UMRS 945, IFR 113, Hôpital Pitié-Salpêtrière, Paris, France; 6 Université Pierre et Marie Curie, ER1 DETIV, Hôpital Pitié-Salpêtrière, Paris, France; University of Cape Town, South Africa

## Abstract

**Background:**

The evolution of HIV-1 and its immune escape to autologous neutralizing antibodies (Nabs) during the acute/early phases of infection have been analyzed in depth in many studies. In contrast, little is known about neither the long-term evolution of the virus in patients who developed broadly Nabs (bNabs) or the mechanism of escape in presence of these bNabs.

**Results:**

We have studied the viral population infecting a long term non progressor HIV-1 infected patient who had developed broadly neutralizing antibodies toward all tier 2/3 viruses (6 clades) tested, 9 years after infection, and was then followed up over 7 years. The autologous neutralization titers of the sequential sera toward *env* variants representative of the viral population significantly increased during the follow-up period. The most resistant pseudotyped virus was identified at the last visit suggesting that it represented a late emerging escape variant. We identified 5 amino acids substitutions that appeared associated with escape to broadly neutralizing antibodies. They were V319I/S, R/K355T, R/W429G, Q460E and G/T463E, in V3, C3 and V5 regions.

**Conclusion:**

This study showed that HIV-1 may continue to evolve in presence of both broadly neutralizing antibodies and increasing autologous neutralizing activity more than 10 years post-infection.

## Introduction

The real impact of the humoral response to the human immunodeficiency virus type 1 (HIV-1) in the course of infection is still a matter of debate [1]–[4]. To date, studies have focused on the neutralizing response since neutralizing antibodies (Nabs) are usually deeply involved in protection against viral infections [5], [6]. In the context of HIV-1 infection, Nabs appear at the early stage of the infection in most of the patients but have been described as ineffective on the long term since they do not seem to be associated with control of viral replication and disease progression [7]. However, Nabs exert a selective pressure on the viral population, leading to continuously evolving viral variants that escape neutralization [8]–[10]. The initial neutralizing antibody response is primarily narrow in its spectrum in most individuals (i.e. autologous neutralization), with heterologous neutralizing antibodies produced in only a fraction of infected individuals later in infection [4], [11], [12]. Several years after primary infection, only a limited percentage of HIV-1 infected patients are able to develop broadly Nabs (bNabs) albeit it appeared that there was a lack of effect of bNabs on disease progression [11], [13], [14]. Some of these patients were characterized as elite neutralizers due the exceptional breadth and potency of their antibodies [15]. A few human broadly neutralizing monoclonal antibodies have been isolated from such patients [16]–[19] and have been shown to be protective in non-human primate studies [20]–[23]. Therefore, the identification of the epitopes targeted by these bNabs, either monoclonal or polyclonal present in human sera, is of prime importance in the perspective of developing an efficient HIV vaccine able to induce protective antibodies [24], [25].

The viral evolution and immune escape experienced by the virus during the acute/early phases of infection have been analyzed in several studies [9], [10], [26]–[30]. These studies have documented the antibody response raised early in infection against the transmitted/founder viruses, the preferentially transmitted variants being considered as those towards which a protective response should be induced by an hypothetical efficient vaccine [31]–[35]. They showed that the pathway that HIV-1 uses to escape the early autologous neutralizing response is not unique, ranging from single amino-acid changes to larger deletions/insertions, and is frequently associated with modification of N-glycosylation sites (PNGS) that led to the concept of an evolving glycan shield at the surface of the envelope spikes [10], [36]. In contrast, little is known about the long-term evolution of the virus in patients who developed bNabs, in particular the mechanism of escape if HIV-1 continues to replicate in presence of these bNabs [37]–[39]. However, this knowledge is crucial for understanding HIV escape to the most efficient Nabs, and might be useful to designing an efficient HIV vaccine. In the present study, we have studied the viral population infecting a long term non progressor (LTNP) HIV-1 infected patient who had developed bNabs at a level compatible with an elite neutralizer status after at least 8 years of infection, over 7 years of follow-up. We provide evidence of continuous evolution of HIV-1 albeit the presence of bNabs, and describe the molecular characteristics of this evolution.

## Materials and Methods

### Ethics Statement

The institutional review board of Pitié Salpêtrière Hospital (Paris, France) approved the study protocol, and each patient enrolled in the French LTNP cohort (ALT ANRS CO15) provided written informed consent.

### Nucleic Acid Extraction, Cloning and Sequencing

Genomic DNA was extracted from PBMC using the QIAamp DNA Blood Midi kit according to the manufacturer’s instructions (Qiagen, Courtaboeuf, France). A 1276 bp *env* fragment encompassing most of the gp120 coding sequence (from upstream of variable region 1 [V1] to downstream of V5) was amplified by nested polymerase chain reaction (PCR) using subtype B *env* -specific primers as previously described [40]. The PCR products were inserted into pCR2.1 (Topo TA cloning kit; Invitrogen, Paisley, UK) and sequenced as described [40]. Sixty-nine clones were obtained (Table 1). The assigned accession numbers were EF179881 through EF179896 and JN634878 through JN634933.

**Table 1:** Description of the samples and molecular characteristics of the *env* clones.

Year	VL (cp/mL)	CD4^+^ cells(cells/mm^3^)	EnvclonesNb	Pseudotyped viruses Nb	Length (Nb AA)		PNGS Nb	dN %	dS %	dN/dS ratio			Evolutionary divergence	Net charges	
					Gp120	Variable loops				Global	Constant regions	Variable regions		Gp120	Variable loops
1996 (T0)	56000	1040	13	0	405.2 (403.6–406.9	137.9 (136.4–139.5)	25.2 (24.5–25.8)	4.1 (3.8–4.4)	5.4 (5.0–5.9)	0.9 (0.8–1.1)	0.8 (0.6–1.1)	1.0 (0.8–1.2)	0.05 (0.05–0.06)	5.4 (4.2–6.7)	2.1 (1.0–3.2)
1997 (T1)	48000	947	14	2	403.1 (401.6–404.6)	135.6 (134.1–137.1)	24.6 (24.1–25.1)	4.3 (4.0–4.6)	5.4(5.0–5.9)	0.8 (0.7–0.9)	1.0 (0.8–1.3)	0.7 (0.6–0.8)	0.08 (0.06–0.09)	1.3 (−0.4–3.0)	−0.1 (−1.8–1.5)
1998 (T2)	76000	888	16	3	405.0 (403.6–405.4)	137.7 (136.3–139.1)	24.9 (24.4–25.1)	4.5 (4.0–4.6)	5.2 (4.8–5.6)	0.9 (0.8–1.0)	1.5 (1.2–1.9)	0.8 (0.6–0.9)	0.06 (0.06–0.07)	4.5 (3.2–5.7)	1.0 (0.2–1.7)
1999 (T3)	112767	870	8	1	403.9 (401.6–406.1)	136.6 (134.3–139.0)	25.0 (24.2–25.8)	4.8 (4.4–5.2)	6.1 (5.4–6.8)	1.0 (0.9–1.2)	1.7 (1.2–2.3)	1.0 (0.8–1.2)	0.07 (0.06–0.08)	6.4 (5.0–7.8)	2.8 (1.3–4.3)
2000 (T4)	102644	694	3	2	404.0 (396.5–411.5)	136.7 (130.4–142.9)	24.7 (21.8–27.5)	3.7 (3.0–4.4)	5.2 (4.0–6.4)	0.8 (0.6–1.0)	0.5 (0.3–0.9)	0.8 (0.5–1.0)	0.08 (0.07–0.09)	2.3 (−5.3–9.9)	−0.1 (−6.7–6.5)
2003 (T7)	852	849	15	5	404.5 (403.3–405.8)	136.9 (135.6–138.1)	25.2 (24.7–25.7)	6.2 (5.9–6.5)	7.7 (7.2–8.2)	0.8 (0.7–0.9)	0.7 (0.5–0.9)	0.7 (0.6–0.8)	0.11 (0.10–0.12)	5.0 (3.5–6.4)	3.3 (2.1–4.6)

dN and dS values result from comparisons between *env* sequences from a given time-point to sequences from previous one. Mean values and ninety-five percent confidence intervals (CI 95% in brackets) are shown. Estimates of evolutionary divergence over sequence pairs relative to the virus population at enrollment (T0). VL: viral load; cp/mL: copies/mL. Nb AA: number of amino-acids.

### *Env*-pseudotyped Virus Construction

**Figure 1:**
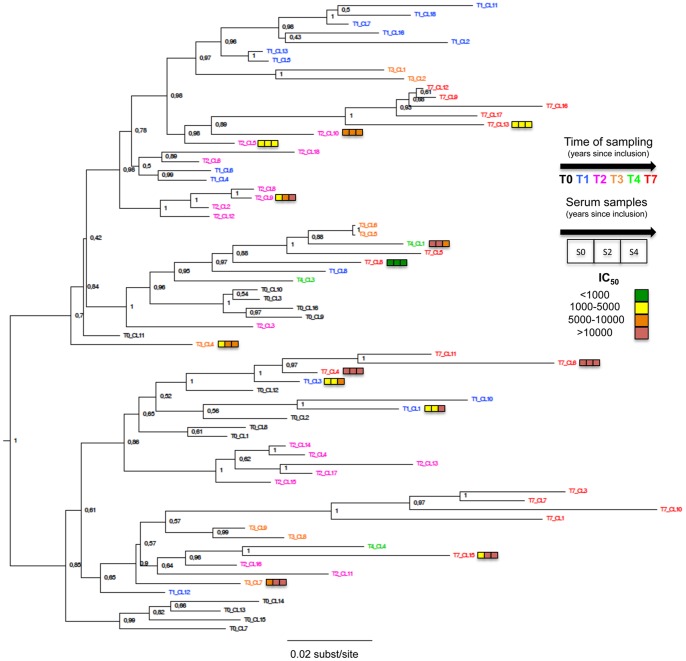
Rooted phylogeny of theHIV-1 *env* sequences (gp120) obtained from PBMC of patient 05005. Codes of the different variants are colored depending of time of sampling. Colored squares represent IC_50_ range of the thirteen pseudoviruses to the three sequential sera (from left to right: S0, S2, S4). For clarity, color code was established: (green) IC_50_<1,000; (yellow) 1,000< IC_50_<5,000; (orange) 5000< IC_50_<10000; (red) IC_50_>10,000. Node labels indicate the Bayesian posterior probability of the supported branch. Scale bar is expressed as number of nucleotides substitutions/site.

We selected 24* env* clones considered to be representative of the viral genetic diversity present at each sampling time-point among the 69 clones. The strategy to generate *env*-pseudotyped viruses expressing gp120 from each clone was previously described [41], [42]. We used the pCR2.1 vector containing the entire NL4.3* env* gene inserted at the EcoRI site. In a first step, the NL4.3* env* gene was digested using NdeI and MfeI (New England Biolabs) and the deleted fragment was replaced by each of the corresponding 24* env* selected clones. Then, chimeric *env* genes were cloned into the EcoRI site of the pCI expression vector (Promega). *Env*-pseudotyped viruses were generated by cotransfecting 3×10^6^ 293T cells using 4 µg of each pCI-env plasmid and 8 µg of pNL4.3.LUC.R_E_ [43] with the FuGene-6 HD transfection reagent (Roche Applied Science, Indianapolis, IN). Viral supernatants were collected 48 h later and purified by filtration (0.45 µM filter) and stored as aliquots at −80°C. Viral infectivity was monitored by infection of 10,000 TZM-bl cells with 100 µL of serial 5-fold dilutions of the viral supernatants in quadruplicate in the presence of 30 µg/mL DEAE-dextran. After 48 h of incubation, infection levels were determined by measuring the luciferase activity of cell lysates using the Bright-Glo luciferase assay (Promega) and a Centro LB 960 luminometer (Berthold Technologies) [44]. Results with relative light unit (RLU) values >2.5 times the negative control (blank pCI vector) were considered positive. Thirteen of the 24 pseudotypes viruses were infectious, and then used for neutralization studies (Figure 1).

### Neutralizing Assays

Patient `05005’s serum was among the very rare samples from LTNPs at entry in the cohort that was able to neutralize (IC_90_≥10) all four primary isolates (FRO, GIL, MBA and KON) of four different clades (B, F, CRF01_AE and CRF02_AG, respectively), selected for their low sensitivity to neutralization [13]. Breadth of neutralization was further assessed toward 6 heterologous primary isolates with top ranking neutralization resistance properties. These subset included 92BR020, 94UG103, 93IN905, and 92TH021 (clades B, A, C and CRF01_AE, respectively), four viruses identified as indicators for cross-clade elite neutralization [15]. The two other viruses were BIG (B) and 92RW020 (A), two moderately resistant viruses [15], [45].

Heterologous neutralization was tested in duplicate using 3-fold serial dilutions (from 1∶20 to 1∶540) of the serum samples. Autologous neutralization experiments were carried out in duplicate using 3-fold serial dilutions (from 1∶40 to 1∶87480) of the serum samples. Briefly, aliquots of 50 µL of the virus dilution (either primary isolate for heterologous neutralization or pseudotyped viruses for autologous neutralization), corresponding to 100 50% tissue culture infectious doses (TCID_50_), were incubated for 1 h at 37°C with 11 µL of each dilution of heat-inactivated sera. The mixture was then used to infect 10,000 TZM-bl cells [46], [47] in the presence of 30 µg/mL DEAE dextran. Infection levels were determined after 48 h by measuring the mean value of luciferase activity of cell lysates. The IC_50_ was determined as the reciprocal serum dilution required to reduce RLUs by 50%.

Neutralization sensitivity of the pseudotyped viruses to the broadly mabs b12, 2G12, PG9 and PG16 was tested in duplicate with 3 fold serial dilutions of each mab, starting from 50 µg/mL (b12 and 2G12) or 10 µg/mL (PG9 and PG16), and following the same procedure.

### Sequences Analyses

Sequence alignments were performed using the MAFFT multiple-alignment software [48]. Amino acid positions were identified according to HxB2 numbering. The number of potential N-glycosylation sites (PNGS) (NX[ST] pattern, where X can be any amino acid) was determined with the N-Glycosite tool [49]. Non synonymous substitutions (dN) rates, synonymous substitutions (dS) rates and dN/dS ratio within each timepoint for either global sequences or constant and variable *env* regions only were calculated with the HyPhy software using SLAC analysis (http://www.datamonkey.org/; [50]). Positive selection, neutral evolution and purifying selection were indicated by dN/dS >1, dN/dS  = 1 and dN/dS <1 respectively. The average evolutionary divergence between sampled sequences and the reconstructed ancestor sequence was estimated using TreeStat v1.6.1 (http://tree.bio.ed.ac.uk/software/treestat/). Analysis of viral diversity among the sixty-nine sequence**s** was performed using the Entropy-One software (http://www.hiv.lanl.gov/content/sequence/ENTROPY/entropy_one.html). Net charges of gp120 were calculated by counting the overall number of charged amino-acids residues per sequence, considering R and K = +1, H = +0.293, D and E as −1. The sequences were screened for recombination breakpoints using the genetic algorithm for recombination detection (GARD) [51]. Positively selected codons were identified using Bayes Empirical Bayes (BEB) analysis implemented in the program codeML, from the PAML package [52]. To detect selection, alignments were fitted to the NSsites models M7 (neutral model, codon values of dN/dS fit to a beta distribution, dN/dS>1 disallowed) and M8 (positive selection model, similar to M7 but with an extra class of dN/dS>1 allowed). Likelihood ratio tests were performed to assess whether permitting codons to evolve under positive selection gives a significantly better fit to the data (model comparisons M7 *vs* M8). To maintain confidence in the inferred positively selected codons, we considered only identified codons with a cut-off posterior probability of 95%.

### Phylogenetic Analysis

The phylogenetic relationship of the 69 *env* sequences was reconstructed using several phylogenetic algorithms [24], [53]. We first reconstructed the sequences phylogeny by maximum likelihood inference, using the program PhyML implemented in the SEAVIEW package, under the General Time reversible (GTR) model of nucleotide substitution. Confidence in the neighbor joining tree topology with ML distances under a GTR model was assessed by 1000 bootstrap replicates with SeaView Software version 4.2.11 [54]. The influence of convergent evolution due to selective pressure on the tree reconstruction was assessed by repeating the analysis with third codon positions only (417 nt), and after removing 33 codons identified as positively selected. Following the identification of 2 recombination breakpoints along the gene sequence, using the software GARD from the HyPhy package [51], phylogenies were also reconstructed after partitioning the alignment.

In order to estimate divergence times of the viral variants, Bayesian MCMC dated phylogenies were reconstructed using the BEAST package [55]. The analysis was conducted under the best-fitting set of molecular, coalescent and demographic models, as indicated by a Bayes Factor >20. These comprised the SRD06 model of nucleotide substitution with separate HKY with gamma-distributed rate heterogeneity at 1^st^ +2d and 3^rd^ codon positions, an uncorrelated lognormal molecular clock model and a Bayesian skyline plot tree prior. Tip dates were informed with the sampling date of the sequences. The BEAST analyses were run for 50 million steps, trees sampled every 100^th^ generation, and a maximum clade credibility tree was selected with the software TREEANNOTATOR, after a 10% burn in. Trees were visualized and edited in FIGTREE v.1.3.1. Considering the recombination breakpoints, we also performed the Bayesian evolutionary analysis after partitioning the subregions separetely, with linked substitution and clock models, but unlinked partition trees.

### Statistical Analysis

Statistical analyses were performed using the PRISM 5.04 Graphpad software package. Evolution of *env* glycosylation, net charges, length of gp120, and length of variable loops were analyzed using a Pearson correlation test. Neutralization potencies of the three successive sera were compared by One-way ANOVA test for repeated measures. The evolution of positively selected amino-acids over time was assessed by linear regression analysis. Changes in viral divergence from the founder virus were analyzed using a Spearman correlation test.

## Results

### Patient: Clinical and Biological History

Patient 05005, infected by a subtype B virus, was enrolled in the French LTNP cohort (ALT ANRS CO15) in 1996. Briefly, inclusion criteria in this cohort were HIV-1 seropositivity for at least 8 years, stable CD4^+^ T-cell count (600 cells/mm^3^ over the previous 5 years), no clinical symptoms, and no antiretroviral therapy. Patient 05005 was selected for the study because of the presence of bNabs at entry in the cohort in a previous study [40], [13]. This patient had been followed up regularly several years after enrollment, and before being treated. HIV-1 viral load (VL) and CD4^+^ T cell counts during the 7 years follow-up are presented in table 1. A regular decrease in CD4+ T cells and a slight but regular increase of VL led to the introduction of antiretroviral therapy (ART) in November 2001, 5 years and 9 months after enrollment in the cohort. Stored peripheral blood mononuclear cells (PBMC) were available at 6 time-points: 1996 (T0), 1997 (T1), 1998 (T2), 1999 (T3), 2000 (T4), and 2003 (T7). They were used for DNA extraction and amplification of *env* sequences. Stored serum samples were available at 3 time-points: 1996 (T0), 1998 (T2), and 2000 (T4). The latter were used for analysis of the neutralizing antibody activity.

### Cross-clade Neutralizing Activity

We previously observed that serum from patient 05005 at time of entry into the ANRS CO15 cohort (T0, 1996) neutralized four primary isolates of four different HIV-1 clades selected based on their low sensitivity to neutralization [40], [13]. These previous neutralization experiments were carried out using the P4P cell line (HeLa-CD4^+^-CXCR4^+^-CCR5^+^ cells) using an immunostaining method for titration [45]. We have confirmed that this serum collected at entry also neutralized additional primary isolates with low sensitivity to neutralization, including four tier 2/3 reference strains identified as indicators for cross-clade elite neutralization [15], [56]. These results were confirmed at T2 with similar neutralization titers against these additional strains (Table 2). Serum at T4 that was available in larger amounts allowed to test the neutralizing activity of patient 05005 toward ten indicator strains using the same assay. All ten primary isolates, representing viruses of six clades (A, B, C, F, CRF01_AE, and CRF02_AG) were neutralized with IC_50_ ranging from 55 to >540 (Table 2), suggesting that patient 05005 presented a profile of elite neutralizer.

**Table 2:** Cross-neutralizing activity of sera from patient 05005.

Serum	Year	Clade B			Clade A		Clade C	Clade F	CRF01_AE		CRF02_AG
		FRO	BIG	92BR020	94UG103	92RW020	93IN905	GIL	MBA	92TH021	KON
T0	1996	Pos	NA	>540	207	285	>540	Pos	Pos	>540	Pos
T2	1998	NA	NA	>540	349	357	>540	NA	NA	>540	NA
T4	2000	>540	>540	>540	153	315	>540	>540	206	>540	55

Values correspond to IC_50_. NA: not available. POS: presence of Nab identified previously using a different assay [13], [36].

### Autologous Neutralizing Activity Against *env* pseudoviruses

The autologous neutralizing activity was studied using pseudotyped viruses expressing selected gp120s. Sixty nine clones of the *env* gene (1276 bp fragment encompassing most of the gp120 coding sequence) were obtained from the PBMCs collected during the follow-up period (Table 1). Starting from 24 gp120*-env* clones representative of the viral population’s diversity at the different time-points, we obtained thirteen *env* pseudoviruses that were infectious (Table 1; Figure 1). The autologous neutralizing activity of the three available sequential sera was tested toward the 13 *env* pseudoviruses. The *env* pseudoviruses displayed a broad range of sensitivity to the autologous sera (Table 3). The most resistant *env* pseudovirus was CL8 of T7 (T7-CL8), suggesting that T7-CL8 represented a late emerging variant that escaped the earlier neutralizing response. Several late clones were however highly sensitive to neutralization by antibodies present in earlier samples (T7-CL4 and T7-CL6). Interestingly enough, we observed a regular increase of neutralization potency of the sequential sera toward 6 *env* pseudoviruses (T1-CL1, T1-CL3, T2-CL9, T3-CL7, T4-CL3, T7-CL15) whereas the neutralization titers were stable overtime for the others (Table 3). When aggregating the data, the mean neutralization titers significantly increased from 6722 [95% CI: 2746–10697] to 10233 [95% CI: 3336–17130] and 15065 [95% CI: 5911–24219] from T0 to T2 and T4, respectively (p = 0.039, One way ANOVA test). This suggests that the autologous neutralizing response continues to increase when HIV continues to replicate, even in patients who already developed broadly neutralizing antibodies.

**Table 3:** Autologous neutralizing activity of sera from patient 05005.

Serum	Year	1997(T1)		1998(T2)			1999(T3)	2000(T4)		2003(T7)					Mean IC_50_ (CI95%)
		CL1	CL3	CL5	CL9	CL10	CL7	CL1	CL3	CL4	CL6	CL8	CL13	CL15	
T0	1996	3099	3142	1684	2748	7477	9000	14880	4073	22561	13382	430	1548	3357	6722 (2746–10697)
T2	1998	3040	2265	1598	6692	6490	43066	13222	5276	17704	16422	869	3440	12940	10233(3336–17130)
T4	2000	17989	5229	1514	22217	7551	56216	7703	8927	21398	16025	643	2510	27924	15065 (5911–24219)
Mean IC_50_		8043	3545	1599	10552	7173	36094	11935	6092	20554	15276	647	2499	14740	

The autologous neutralizing activity was evaluated toward thirteen pseudoviruses issued from 5 time-points (1997 to 2003). Values correspond to IC_50_ serum titers. Mean IC_50_ is indicated for each clone. Mean IC_50_ and 95% confidence interval (CI 95%) are indicated for each serum.

### Sensitivity of the *env* pseudoviruses to Human Broadly Neutralizing Monoclonal Antibodies

Sensitivity to neutralization of the 13 *env* pseudoviruses was analyzed using the following monoclonal antibodies (mabs): b12, 2G12, PG9 and PG16. A wide spectrum of sensitivity was observed for each mab, without any clear evidence for temporal relationship (Table 4). Clones with low sensitivity to b12, 2G12 and PG9 were already present in the early samples (T1 and T2). However, two clones that appeared as the most resistant to the neutralizing mabs tested (resistant to 2G12, PG9 and PG16) were isolated from the late sample (T7-CL6 and T7-CL15). This observation would need to be further studied using a larger number of clones in order to get stronger data concerning a possible evolution of the quasispecies toward increased resistance to broadly active mabs. The 13 envelope sequences did not carry any specific mutation in seven sites that have been previously shown to impact b12 sensitivity [24]. Similarly, neither loss of potential N-glycosylation sites (PNGS) at positions 156, 160 and 173 nor single mutations previously shown to impact PG9 and PG16 sensitivity were observed among the 13 variants [57]–[59]. In contrast, the three *env* pseudoviruses that were 2G12-resistant (IC_50_>50 µg/mL) did not harbor the PNGS at position N392 known to be essential for the 2G12 epitope [26], [42], [60], [61].

**Table 4:** Sensitivity to neutralization by human broadly neutralizing monoclonal antibodies of the Env clones issued from patient 05005.

	IC_50_ (µg/mL)			
	b12	2G12	PG9	PG16
**CL1T1**	13.86	11.40	0.02	0.02
**CL3T1**	0.21	1.35	1.80	0.11
**CL5T2**	2.88	14.10	1.02	0.12
**CL9T2**	0.11	0.36	>10	0.26
**CL10T2**	<0.02	0.62	>10	0.34
**CL7T3**	<0.02	>50	0.91	0.28
**CL1T4**	>50	1.85	0.07	0.02
**CL3T4**	0.15	0.90	>10	2.67
**CL4T7**	<0.02	1.58	0.28	0.08
**CL6T7**	0.07	>50	>10	>10
**CL8T7**	1.29	1.72	0.71	0.07
**CL13T7**	0.16	14.5	6.52	0.27
**CL15T7**	0.02	>50	>10	>10

### Phylogenetic Analyses

**Figure 2:**
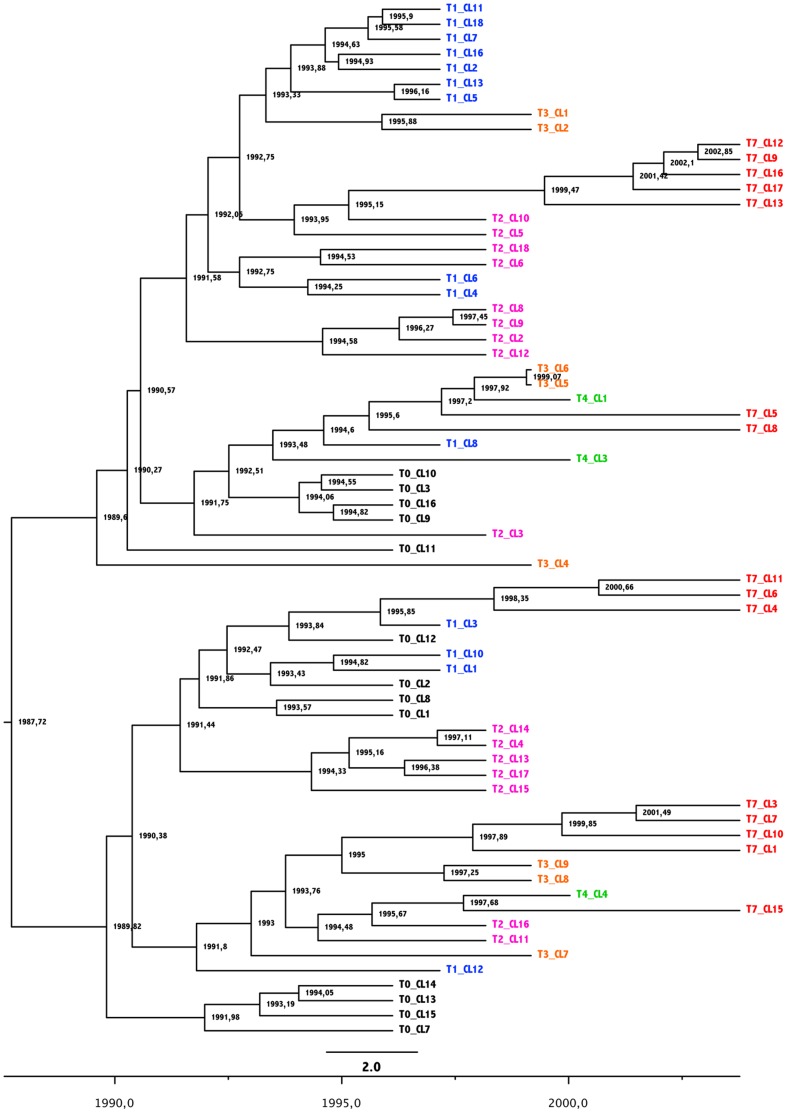
Time-scaled phylogenetic tree of HIV-1 *env* sequences (gp120) obtained from PBMC of patient 05005. Codes of the different variants are colored depending of time of sampling. X axis figures time as measured in years before the most recently sampled patient sequence. Branch nodes indicates estimated dates.

We used Bayesian Markov Chain Monte Carlo (MCMC) inference to reconstruct the phylogeny of the viral population found in patient 05005 and estimate divergence times within the viral population. Since two recombination breakpoints were identified in the entire set of the sixty-nine sequences (in the C2 region, from amino acids positions Y191 to P220 [*HxB2 numbering*] and the C3 region from amino acids positions N340 to K362), we have analysed the three sub-regions independently, and as well as together. Although some differences in the branching patterns of the reconstructed trees were observed, we found concordant estimates between the partitioned data and global sequences for both the time of the most recent common ancestor (tMRCA) and mean rate of evolution of the population (data not shown). The tMRCA of the 69 *env* sequences was estimated around September 1987 (95% Highest probability Density [HPD]: December 1982-November 1991). This result was concordant with the patient’s history, enrolled in the LTNP cohort in 1996 after a minimum of 8 years of infection. The estimated mean rate of evolution of the viral population was 6.9×10^−3^ nucleotide substitutions per site per year [95% HPD: 4.81×10^−3^–9.38×10^−3^). Our estimate is also consistent with previous rates of intra-host HIV-1 evolution [62]. The reconstructed phylogenies showed that the 69* env* clones form two distinct phylogenetic clusters diverging from the root, suggesting either the initial transmission of two closely related variants or an early selective process resulting in two co-dominant variants (Figure 1 and 2). The phylogeny shows little chronological organization. Sequences sampled at a given time point did not form monophyletic clusters, but rather emerged from pre-existing populations throughout the course of the infection (Figure 2). For instance, some clones sampled at T7 (i.e. T7-CL4, T7-CL6 and T7-CL11) shared a common ancestor with viruses sampled at T1 (i.e. T1-CL3), while others (i.e. T7-CL9, T7-CL12, T7-CL13, T7-CL16 and T7-CL17) clustered with clone CL10 sampled at T2 (67 months earlier). This is suggestive of continuous re-emergence of variants from viral reservoirs. Since the data were generated from proviral DNA, sequences could be representative of either circulating Envs at the time of sampling or archived proviral sequences. However, the fact that the sequences collected at the last time point (T7) were located on the longer branches of the tree, indicative of a longer evolution, suggests that most of the sequences were representative of circulating variants at the time of sampling (Figure 1).

### *Env* Evolution during the Follow-up

**Figure 3:**
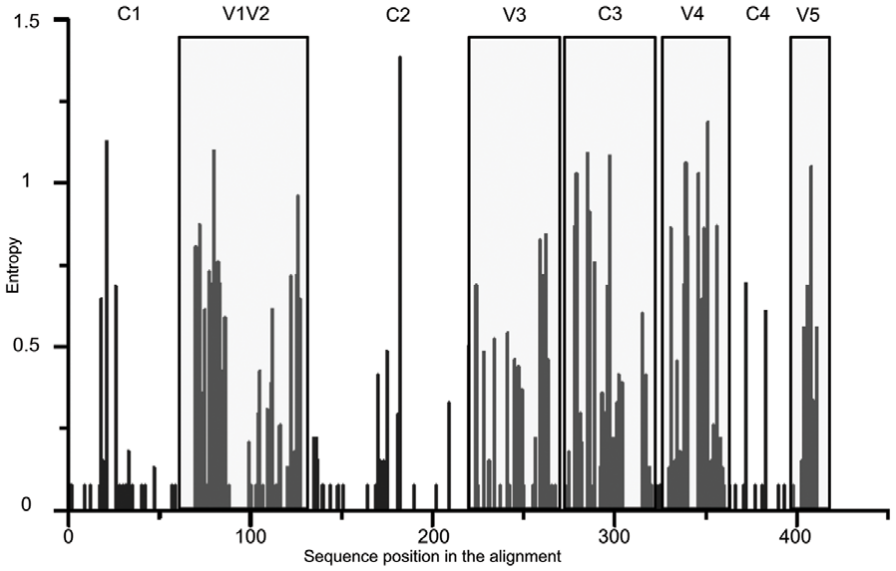
Entropy plot of the 69 *env* sequences. Evaluation of the amino-acid variations among the 69 clones over the gp120 envelope. Constant and variable regions are indicated on top. Variable regions are highlighted in light grey. C3 constant region is also highlighted in light grey.

The estimated viral divergence over the 7 years of follow-up showed that viral diversification continued in patient 05005 at the chronic stage of infection. Based upon the mean number of substitutions per site, we observed a continuous increase of viral divergence through time ranging from 0.054 (95% CI: 0.049–0.06) substitutions/site at T0 to 0.108 (0.099–0.116) substitutions/site at T7 (Table 1; Spearman r test  = 0.97, p  = 0.017). Previous studies reported a global increase in gp120 length over time, particularly in variable regions, and in the number of PNGS during the course of infection, suggesting that these mechanisms support escape to neutralizing antibodies [10], [63], [64]. In our study, neither the length of both gp120 and variable regions, nor the number of PNGS increased during the 7 years follow-up (Table 1). Similarly, we did not observe any significant net charges in amino acid substitution over the course of infection, considering either the entire gp120 sequences or the variable regions (Table 1). Analysis of viral diversity among the sixty-nine sequence**s** showed that the variable regions as well as the C3 region were highly variable among the 69 clones, mean entropy varying from 0.063 (95CI: 0.043–0.083) in constant regions to 0.307 (95% CI: 0.281–0.334) in variable regions, with an intermediate value (0.230; 95% CI: 0.173–0.286) in the C3 region (Figure 3).

We tried to identify selective pressures that may have shaped genetic variation among all clones. dN/dS ratios from the successive time points were calculated for each constant and variable region. Except for the last time point, non synonymous (N) and synonymous (S) substitution rates did not increase over time, varying respectively from 4.1% to 4.8% and 5.2% to 6.1% of the overall rate of substitutions. Clones isolated at T7 presented a significant higher diversity, considering both N (6.2%) and S (7.7%) substitutions. When considering the full length gp120 sequences, we have found no evidence of clear positive selection (expressed as dN/dS >1), irrespective of the time point (mean dN/dS varying from 0.79 at T4 to 1.02 at T3).

**Figure 4:**
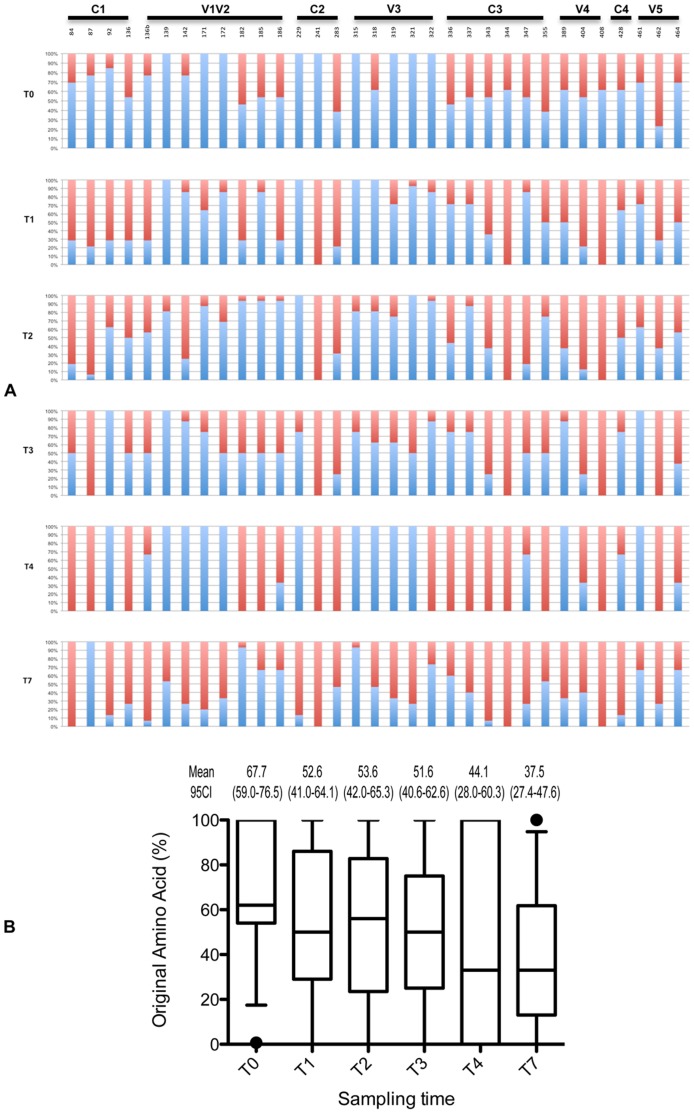
Evolution of the 33 positively selected codons over time. (A) Each bar represents the percentage of original dominant AA (in blue) and the percentage of variant AA (in red). Positions refer to HxB2 numbering. (B) Evolution of the percentage of original AA over time. Results are shown as distribution of these percentages for each time point. For each distribution, the horizontal lines represent the 10^th^, 25^th^, 50^th^ (median), 75^th^ and 90^th^ percentiles. Mean value and 95% confidence intervals (CI 95%) are indicated at the top of each box.

The evolution of gp120 was illustrated by numerous single amino acids (AA) substitutions both in variable and constant regions. Among these numerous substitutions, we identified 33 positively selected codons using the Bayes Empirical Bayes (BEB) analysis implemented in codeML (cut-off posterior probability P>95%; Figure 4). These results were confirmed with a model-test comparison: M8 (positive selection model) had a significantly better fit to the data than M7 (neutral model) (p<0.05). Positive selection pressure was observed both in variable and constant regions (particularly C3), suggesting that these regions are probably targets of the adaptative immune response. Considering AA substitutions among the 33 positively selected codons, we showed a continuous evolution overtime, even at the last time-point. Figure 4A illustrates the ratio of original AA (in blue) and variant AA (in red) at the different time-points at each of these positions. For instance, it shows that the variant AA at position 84 in C1 that was present in 30% of clones at T0 was found in 100% of clones and T4 and T7. A synthetic analysis of the evolution at the 33 positions is shown in figure 4B which indicates that the percentage of original AA at these positions decreased regularly overtime, from 67.7% to 37.5% from T0 to T7 (Figure 4B, R ^2^ = 0.85). If some (5/33) AA substitutions observed during the follow-up reverted, most did not and became dominant (20/33). Of note is the fact that the 3 substitutions that were found in all the clones of the two late samples (T4 and T7) were located in constant regions (V84I in C1, K241N in C2 and R344Q in C3), suggesting that they might be associated with a biological advantage.

### Phylogenetic Analysis, Selective Pressure and Neutralizing Activity

Trying to link the phenotypic properties to the genotypic properties, we analyzed whether the level of sensitivity to autologous neutralization of each of the thirteen infectious clones would be associated with specific genetic features. We did not observe a strong clustering of sensitive or resistant *env* pseudoviruses in specific branches of the tree (Figure 1).

**Figure 5:**
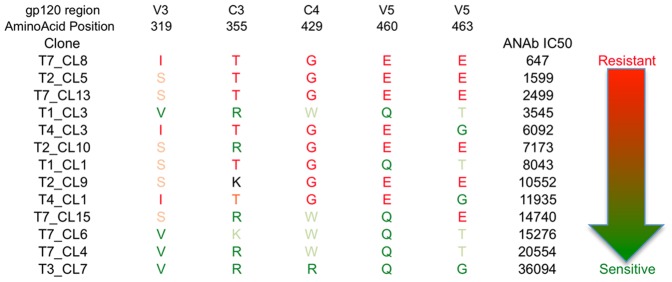
Identification of potential amino-acids signature sequence associated with autologous neutralization phenotype in patient 05005. The sequence includes 5 non-contiguous AA identified as related to autologous Nab activity after pairwise comparison of positively selected amino-acids among thirteen pseudoviruses. Clones are ordered from the most resistant (top) to the least resistant (bottom) in accordance with mean autologous Nab titer. AA associated with sensitivity to autologous neutralization are shown in dark green. AA that differed from the most susceptible but were associated with susceptibility are shown in light green. Conversely, AA associated with resistance to autologous neutralization are shown either in dark red or light red. Numbering positions are relative to HXB2 gp120.

More in depth, we asked whether positively selected AA could be associated to sensitivity to autologous neutralization. Not a single AA substitution by itself clearly predicted a change in sensitivity to autologous neutralization. We therefore tried to identify whether any signature pattern (i.e. any combination of AA substitutions among the 33 positively selected codons) would be associated with sensitivity to autologous neutralization. To that purpose, we ranked the 13 pseudoviruses according to their mean sensitivity to autologous neutralization (Figure 5). Then, we compared the sequence of the most resistant *env* pseudovirus (T7-CL8) to that of the most sensitive *env* pseudovirus (T3-CL7), and we performed successive analyses by adding one by one sequences of the subsequent most resistant *env* pseudovirus (T2-CL5, T7-CL13, …) *versus* the subsequent most sensitive (T7-CL4, T7-CL6, …) *env* pseudovirus. Considering only the 3 most sensitive clones (T3-CL7, T7-CL4 and T7-CL6 with mean IC_50_ range from 15276 to 36094) and the three most resistant clones (T2-CL5, T7-CL8 and T7-CL13 with mean IC_50_ range from 647 to 2499), we identified 5 AA positions that could be associated with sensitivity to autologous neutralization (Figure 5). They were located in V3, C3 and V5 regions. The most sensitive *env* pseudoviruses presented the sequence V^319^R/K^355^R/W^429^Q^460^G/T^463^ whereas the most resistant viruses had the sequence I/S^319^T^355^G^429^E^460^E^463^ (Figure 5). The potential impact of these variations was strengthened by net charges changes for three of them (R/K^355^T, Q^460^E and G/T^463^E).

## Discussion

Recent investigations of the genetic diversity of HIV-1 at transmission and of the evolution of the virus during the early phases of infection have provided critical insights into the immunovirological mechanisms that take place in the earliest stages of HIV-1 infection [31], [33], [35], [65]–[69]. Several studies allowed the identification of adaptive changes that enable escape from immune responses, particularly escape from neutralizing antibodies during the early phases of HIV-1 infection [8]–[10], [70]. They clearly demonstrated the continuous escape to autologous neutralizing antibodies. However, autologous antibodies have a narrow spectrum of neutralization, restricted to the subject’s own virus. While all these studies focused on the early phases of HIV-1 infection, we wanted to document the late, rather than early, evolution –if any- of HIV-1 in presence of broadly cross-neutralizing antibodies. Identifying the mechanism of escape to bNabs would be an additional element useful for vaccine designers. Our study was made possible through the follow-up of a LTNP patient who was identified more than 8 years after seroconversion while having already developed broadly neutralizing antibodies [40], [13]. Based on additional neutralization assays using ten primary isolates of six different clades selected based on their moderate (tier 2) or low (tier 3) sensitivity to neutralization, we first confirmed in the present report that this patient might be considered as an elite neutralizer. Indeed, his serum both at entry in the LTNP cohort, more that 8 years post-infection, and several years later, neutralized all the viruses tested including strains identified previously as indicators of elite neutralization [15].

Sixty-nine *env* clones (gp120 region) were obtained from PBMC DNA at six time points starting from the entry in the cohort in 1996 up to 7 years later. Using Bayesian MCMC inference, we estimated the time of infection of patient 5005 around 1987 (95% CI: December 1982 to November 1991), nine year before entry in the cohort, confirming the recorded clinical history. We therefore estimate that the entire analysis that we performed has corresponded to a sixteen years duration of HIV-1 infection. Evidence of ongoing viral evolution was found in our LTNP patient, supported by both the phylogenetic analyses that showed a continuous diversification and an increasing divergence over time, as it was previously described by Shankarappa et al., albeit on a shorter duration (6–12 years) of follow-up [71].

For the first time to our knowledge, we show in this report that autologous neutralizing activity may still continue to increase over time more than 10 years after infection. This was illustrated by the fact that we observed a regular increase of autologous neutralization potency of the sequential sera toward infectious pseudotyped viruses issued from the various time points. However, due to the polyclonal nature of antibodies present in the serum samples, we do not know whether the neutralizing antibodies that were specific for the patient’s envelopes were those capable of cross-neutralization. Interestingly, the most resistant pseudotyped virus was issued from the last visit sample. This suggests that this variant represented a late emerging escape variant and, additionally, provides phenotypic evidence of ongoing viral evolution approximately 16 years post-infection. Taken together, these data indicate first that HIV-1 continues to evolve *in vivo* more than ten years after infection even in presence of both autologous Nabs and heterologous bNabs. This means that, albeit the presence of cross-clade bNabs that are supposed to target conserved epitope(s), HIV-1 is still able to evolve to bypass this blockade. A similar observation of late continuous evolution and escape was recently reported in a patient who developed bNabs to the CD4-binding site [72]. Secondly, we show that neither a continuous increase of autologous Nabs nor the presence of bNabs seem to allow the control of the patient’s clinical evolution, since a regular increase of virus load and a regular decrease of CD4^+^ T-cell counts led to the introduction of antiretroviral treatment five years after entry in the cohort. Our observation does not support previous findings who suggested that there may be limits to the capacity of HIV-1 to evolve continuously in response to Nabs and that the Nab response may contribute to the long-term control of HIV [3], [39]. Of note also is the fact that variants present at a given time point display a wide diversity in sensitivity to autologous neutralization, as previously reported by others [3].

A wide spectrum of sensitivity to broadly active mabs was observed among the pseudotyped viruses that we generated. Two clones that appeared as the most resistant to the neutralizing mabs were isolated from the late sample, suggesting that further studies using a larger number of clones would deserve to be done to answer the question of a possible intra-host evolution of HIV-1 toward increased resistance to broadly active mabs. Interestingly, these two late clones were resistant to the 3 mabs that target glycan-dependent epitopes.

Many reports have shown that neutralization escape to Nabs is often associated with longer variable loops and increased number of PNGS [10], [32], [63], [64], [73], [74]. We did not observe any significant change over time neither in variable loops length nor number of PNGS in the gp120 sequences obtained from the sequential samples of our patient. It could be argued that in contrast to our study, most of the previous observations were performed in samples collected during the early years post-infection in each case and that these properties may be signatures of early escape. Therefore, other mechanisms may be involved in late neutralization escape. We cannot exclude that such increase in variable loops lengths and number of PNGS occurred in our patient but had reached a plateau before entry in the cohort.

We identified 33 positively selected codons across gp120, including four in C1, three in C2, six in C3 and one in C4. Particularly, the implication of the C3 region was confirmed by the analysis of diversity using Entropy-One software. The role of the third constant region of gp120 in neutralization escape was already emphasized in previous studies, in viruses of both clades B and C [27], [28], [74]–[76]. Acquisition of positively selected AA and their conservation over time suggested their implication in *env* viral escape. In contrast, reversions of single substitutions observed in several positions at the last time-points may be indicative of constraints on the *env* region to maintain viral fitness. Finally, we tried to identify sequence signatures that would be associated with escape to neutralization in our particular case of late escape. Recent studies reported original computational methods useful to define molecular signatures that correlate with neutralization phenotype [24]. They allowed for instance to identify a three-amino-acid substitution pattern in V4 of subtype C viruses that was associated with greater neutralization potency [77]. We therefore tried to identify whether any signature pattern among the 33 positively selected codons would be associated with sensitivity or resistance to autologous neutralization. Not a single AA substitution by itself clearly predicted a change in sensitivity to autologous neutralization. However, we identified five amino acids whose variation may impact neutralization and might be associated with escape to bNabs in our patient. They were located in V3, C3 and V5 regions, and the potential impact of these variations was strengthened by net charges changes for three of them (R/K^355^T in C3, Q^460^E and G/T^463^E in V5).

One limitation of our study is that we analyzed the evolution of the virus based on DNA sequences present in PBMC. It is usually considered that plasma RNA gives a more accurate picture of the viral population at time of sampling than viral DNA, since proviral DNA contains a significant fraction of archived sequences from earlier times. This has been shown in elite suppressors [78], [79] and in patients receiving highly active antiretroviral treatment [80], [81]. However, several studies clearly indicated that sequences obtained at subsequent time points from plasma RNA and PBMC proviral DNA of a given untreated individual form a single virus population at most time points, therefore allowing evolutionary studies of viral sequences from different sources [71], [82], [83]. A second limitation may have been due to the introduction of an antiretroviral treatment in our patient in 2001, two years before the last sample that we explored. However, if this treatment should have slow down the evolution of the virus during the last two years of our follow up, this was not visible through the biological data since the plasma viral load was still detectable in 2003 (T7∶852 copies/mL) and the molecular analyses clearly showed a continuous evolution at T7 (Table 1). A third limitation may be linked to the strategy used to produce the pseudotyped viruses. Indeed, the sequences were not obtained using the single genome amplification technology that is considered as a gold standard to avoid recombination events during the PCR from bulk DNA, and the pseudotyped particles harbored chimeric envelopes composed of each gp120 associated with NL4.3 gp41. Although we cannot exclude that this heterologous association had effects on the sensitivity to neutralization by antibodies directed to gp120, one advantage was that all the chimeric envelopes were generated similarly limiting the variability due to the diversity of gp41 sequences.

### Conclusions

In conclusion, we have shown through the reported case that HIV-1 may continue to evolve in presence of both broadly neutralizing antibodies and increasing autologous neutralizing activity more than 10 years after infection. Such clinical material may provide unique opportunities to reveal the mechanisms and the molecular determinants of escape of HIV-1 to the most potent broadly cross-neutralizing antibodies. Continued study of HIV-1 evolution in similar exceptional situations may help to refine the design of relevant vaccine immunogens.
